# Pathogenic Mechanism of the FIG4 Mutation Responsible for Charcot-Marie-Tooth Disease CMT4J

**DOI:** 10.1371/journal.pgen.1002104

**Published:** 2011-06-02

**Authors:** Guy M. Lenk, Cole J. Ferguson, Clement Y. Chow, Natsuko Jin, Julie M. Jones, Adrienne E. Grant, Sergey N. Zolov, Jesse J. Winters, Roman J. Giger, James J. Dowling, Lois S. Weisman, Miriam H. Meisler

**Affiliations:** 1 Department of Human Genetics, University of Michigan, Ann Arbor, Michigan, United States of America; 2 Life Sciences Institute, University of Michigan, Ann Arbor, Michigan, United States of America; 3 Department of Cell and Developmental Biology, University of Michigan, Ann Arbor, Michigan, United States of America; 4 Department of Neurology, University of Michigan, Ann Arbor, Michigan, United States of America; University of California San Diego School of Medicine, United States of America

## Abstract

CMT4J is a severe form of Charcot-Marie-Tooth neuropathy caused by mutation of the phosphoinositide phosphatase *FIG4/SAC3*. Affected individuals are compound heterozygotes carrying the missense allele *FIG4*-I41T in combination with a null allele. Analysis using the yeast two-hybrid system demonstrated that the I41T mutation impairs interaction of FIG4 with the scaffold protein VAC14. The critical role of this interaction was confirmed by the demonstration of loss of FIG4 protein in VAC14 null mice. We developed a mouse model of CMT4J by expressing a *Fig4*-I41T cDNA transgene on the *Fig4* null background. Expression of the mutant transcript at a level 5× higher than endogenous *Fig4* completely rescued lethality, whereas 2× expression gave only partial rescue, providing a model of the human disease. The level of FIG4-I41T protein in transgenic tissues is only 2% of that predicted by the transcript level, as a consequence of the protein instability caused by impaired interaction of the mutant protein with VAC14. Analysis of patient fibroblasts demonstrated a comparably low level of mutant I41T protein. The abundance of FIG4-I41T protein in cultured cells is increased by treatment with the proteasome inhibitor MG-132. The data demonstrate that FIG4-I41T is a hypomorphic allele encoding a protein that is unstable *in vivo*. Expression of FIG4-I41T protein at 10% of normal level is sufficient for long-term survival, suggesting that patients with CMT4J could be treated by increased production or stabilization of the mutant protein. The transgenic model will be useful for testing *in vivo* interventions to increase the abundance of the mutant protein.

## Author Summary

Charcot-Marie-Tooth disease type 4J is a severe neurological disorder with childhood or adult onset and progression to loss of mobility and death. Patients inherit a mutation that changes amino acid residue 41 of the FIG4 protein from isoleucine to threonine. We report that this mutation destabilizes the FIG4 protein by blocking its interaction with a stabilizing protein partner. We developed a mouse model of CMT4J and found that a low level of expression of the mutant protein, 10% of wildtype level, is sufficient to prevent lethality. This work provides the scientific basis for development of a directed treatment for this rare, lethal disorder.

## Introduction

The lipid phosphatase FIG4/SAC3 is broadly expressed in eukaryotic cells from yeast to mammals. Mutations of *FIG4* are responsible for Charcot-Marie-Tooth Disease type 4J (OMIM 611228), an atypical, autosomal recessive form of CMT with severe motor dysfunction and rapid progression [1], [2]. *FIG4* phosphatase activity specifically removes the 5-phosphate from the inositol ring of PI(3,5)P_2_, a membrane-bound phospholipid that acts as a molecular signal for trafficking and fusion of intracellular vesicles. In yeast, Fig4p is localized to the vacuole membrane in a protein complex that regulates the synthesis and turnover of PI(3,5)P_2_ [3]–[5]. In mammalian cells, the PI(3,5)P_2_ biosynthetic complex is localized in the endosomal/lysosomal vesicle system [6]. Deficiency of mammalian FIG4 or VAC14 leads to accumulation of cytoplasmic vacuoles in tissues and in cultured fibroblasts and neurons [1],[3],[7],[8].

We previously identified a spontaneous null mutant of mouse *Fig4* caused by a transposon insertion [1]. The most striking phenotypes of the *Fig4* null mice are spongiform degeneration of the brain and loss of neurons from the dorsal root ganglia, resulting in a severe movement disorder and lethality between 1 and 2 months of age (see video supplement to [1]). At the cellular level, *Fig4* null fibroblasts exhibit reduced levels of PI(3,5)P_2_ [1], [9], [10]. In the CNS, astrocytes and neurons exhibit accumulation of p62, ubiquinated protein and other autophagic components in cytoplasmic inclusion bodies [11]. These abnormalities demonstrate that PI(3,5)P_2_ is required for completion of basal autophagy, and indicate that there is a defect in resolution of autolysosomes in deficient cells [12].

The biosynthetic complex that regulates PI(3,5)P_2_ contains two major proteins in addition to FIG4, the 5-kinase FAB1/PIKfyve, which phosphorylates position 5 of the inositol ring in PI3P, and the scaffold protein VAC14, composed of multiple heat-repeat structural domains [3]. Stable localization on the yeast vacuolar membrane requires interaction between Fig4p, Fab1p and Vac14p, and loss of one protein results in mislocalization of the other two [3], [9]. In the mouse, the phenotype of the spontaneous *Vac14* mutation L156R mimics the *Fig4* null phenotype, with neurodegeneration, cellular vacuolization and defective autophagy [3], [11]. *Vac14*-L156R is located in a heat repeat domain and the mutation reduces binding affinity for FAB1, thereby disrupting the PI(3,5)P_2_ biosynthetic complex [3]. These mutants in yeast and mouse demonstrate the importance of the stable complex between FIG4, FAB1 and VAC14.

Patients with CMT4J are compound heterozygotes at the *FIG4* locus, carrying the shared missense mutation I41T, on a common haplotype, in combination with a unique or “private” null allele [1]. The frequency of the I41T allele is less than 1/500 in the Northern European population [1]. The corresponding yeast mutant, I59T, retains partial function in a yeast assay for correction of the vacuole phenotype [1], [13]. Disease onset in CMT4J patients with the genotype *FIG4^I41T/−^* may occur in childhood or adult life. The rapid decline of motor function in adult onset patients resembles the course of ALS, and deleterious mutations of *FIG4* have also been identified in patients with ALS [13]. In order to generate a mouse model of human CMT4J, we have expressed a *Fig4* cDNA construct containing the I41T mutation in transgenic mice. Here we report the dose-dependent rescue of the *Fig4* null phenotype by the *Fig4*-I41T transgene. We also demonstrate that the pathogenic mechanism of the I41T allele is based on defective interaction with the scaffold protein VAC14, resulting in destabilization of the FIG4 protein *in vivo*.

While this work was in progress, Shisheva and colleagues reported related work indicating that the short half-life of a GFP-FIG4 fusion protein in cultured cells is increased by over-expression of myc-VAC14, and that the I41T mutation prevents this increase [14]. The authors proposed that VAC14 has a novel regulatory role in the turnover of FIG4 protein [14]. Using the yeast two hybrid system, we demonstrate here that the direct interaction between VAC14 and FIG4 is impaired by the I41T mutation. The reduced interaction results in greatly reduced abundance of FIG4-I41T protein in patient fibroblasts. We further demonstrate that wildtype FIG4 requires VAC14 for stability *in vivo*. Finally, we find that overexpression of mutant I41T protein can compensate for its reduced binding affinity and rescue the mouse model of CMT4J. This work extends the previous observations and advances our understanding of the pathogenic mechanism of the *FIG4*-I41T mutation.

## Results

### Impaired interaction of FIG4-I41T with the scaffold protein VAC14

**Figure 1:**
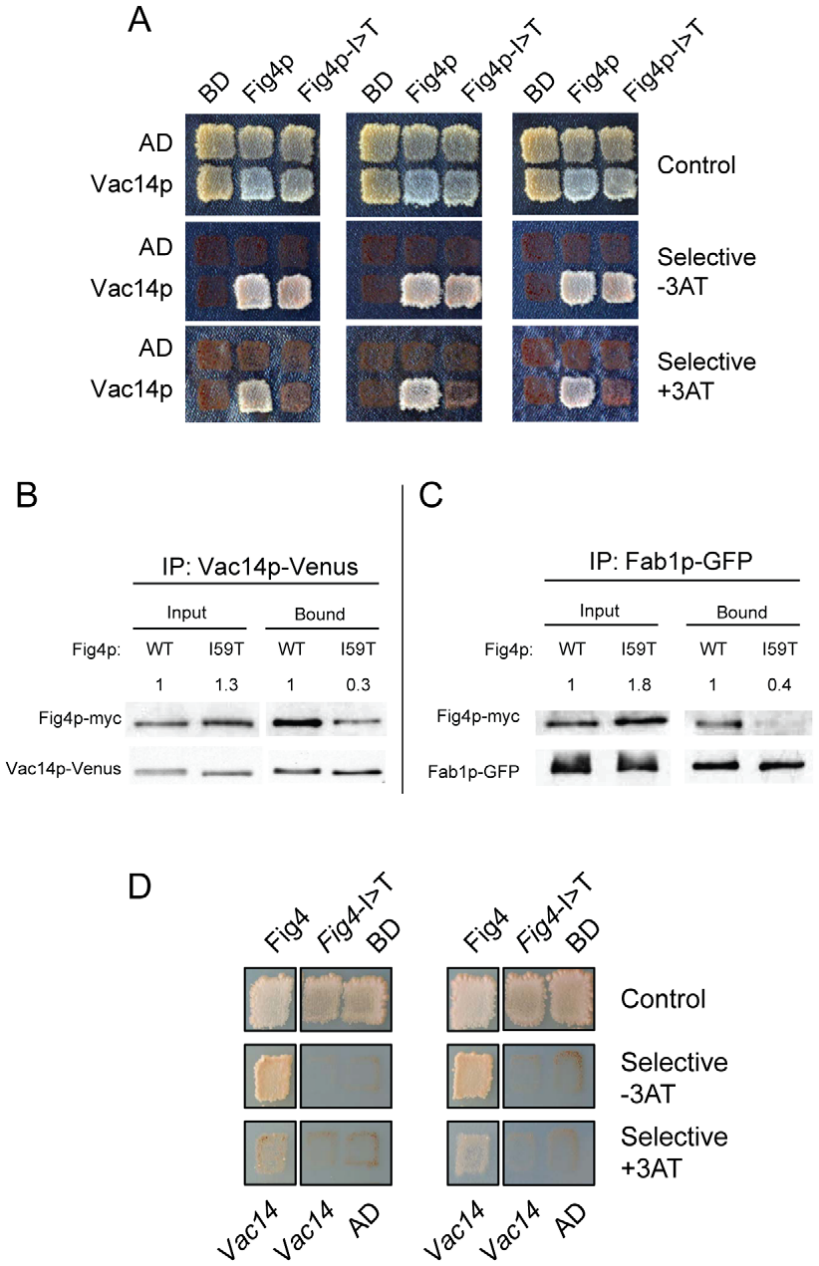
Impaired interaction of FIG4-I41T mutant protein with VAC14 and reduced co-immunoprecipitation with FAB1. A) Directed yeast two-hybrid interaction of wildtype and mutant yeast Fig4p with Vac14p. Selection for Leu and Trp prototrophy was carried out under less stringent (−3AT) or more stringent (+3AT) conditions. B) Co-immunoprecipitation of myc-tagged Fig4p and GFP (Venus) -tagged Vac14p from yeast lysates. C) Co-immunoprecipitation of myc-tagged Fig4p and GFP-tagged Fab1p. D) Directed yeast two-hybrid interaction of human FIG4 and human VAC14. AD, activation domain alone; BD, DNA binding domain alone. The numbers above the lanes in B and C were obtained by densitometry of non-saturated images and represent the ratio of Fig4p∶Vac14p and Fig4p∶Fab1p; the ratio for mutant Fig4p was normalized to the wildtype ratio.

The corresponding yeast mutation, Fig4-I59T, results in impaired vacuole morphology and defective regulation of PI(3,5)P_2_ [1], [13]. We tested the interaction of Fig4p-I59T with the Fig4p binding partners Vac14p and Fab1p using a directed yeast two hybrid assay. Fig4p was fused to the DNA binding domain of GAL4 and Vac14p was fused to the transcription activation domain. The I59T mutant did not support growth under stringent selection in the presence of 3AT (Figure 1A). This result demonstrates reduced binding of the Fig4p-I59T mutant to Vac14p. This result was confirmed in a co-immunoprecipitaton assay using myc-tagged Fig4p and GFP-tagged Vac14p [15]. Wildtype and mutant yeast proteins were expressed at similar levels, but co-precipitation of Vac14p was reduced by approximately 75% for Fig4p-I59T (Figure 1B). Co-immunoprecipitation of GFP-labeled Fab1p was reduced to a similar extent by the Fig4p-I59T mutation (Figure 1C). The latter could be an indirect effect of impaired interaction with Vac14p.

To confirm the effect of the I41T mutation in the context of the mammalian proteins, we tested the interactions of human FIG4 and human VAC14 in the yeast two hybrid system. The I41T mutation significantly impaired the interaction between the two human proteins, preventing growth on both of the selective media (Figure 1D). These experiments demonstrate that the isoleucine-to-threonine substitution reduces the direct interaction of FIG4 with VAC14.

### Interaction with VAC14 is required for stability of wildtype FIG4 protein *in vivo*

**Figure 2:**
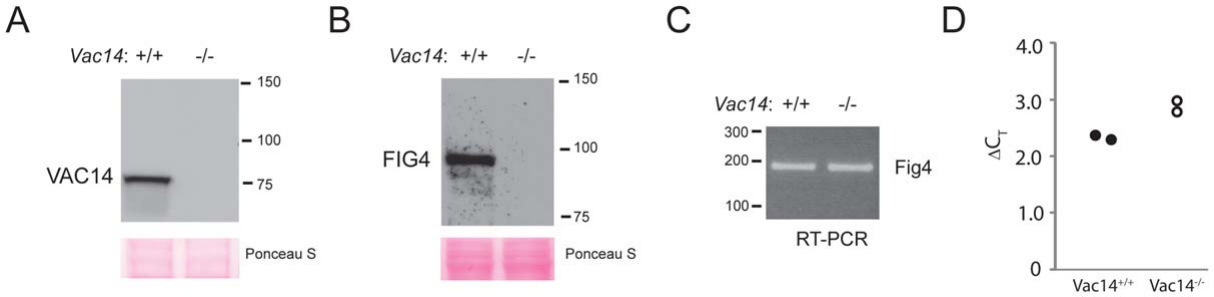
Low level of wildtype FIG4 protein in *Vac14* null mice. A) Absence of VAC14 protein in *Vac14* null mouse. Protein extracts from wildtype and *Vac14* null P0 mice were probed with polyclonal antibody to VAC14 as described [9]. B) Absence of FIG4 protein in *Vac14* null mouse. The same extracts were probed with monoclonal antibody to FIG4. C, D) RT-PCR of RNA from *Vac14* null mice with forward primer in Fig4 exon 3 and reverse primer in exon 4 detects a normal level of *Fig4* transcripts. ΔC_T_, C_T_ for *Fig4* minus C_T_ for *Tbp*.

To investigate the *in vivo* dependence of FIG4 on interaction with the VAC14 scaffold protein, we examined FIG4 levels in tissues from a *Vac14* null mouse [7]. The absence of VAC14 in the null mouse was confirmed by Western blot (Figure 2A). To detect FIG4 protein we generated a monoclonal antibody to a bacterially-expressed 220 amino acid fragment from the C-terminus of FIG4 (Materials and Methods). The monoclonal antibody recognizes a single protein of ∼100 kDa in homogenates of mouse tissues, consistent with the calculated molecular weight of 103 kDa for the 907 amino acid FIG4 protein (Figure S1). Remarkably, the abundance of FIG4 protein was greatly reduced in the *Vac14* null mouse (Figure 2B), although the level of *Fig4* mRNA was normal (Figure 2C, 2D). Although FIG4 protein was undetectable in the tissue extract, a very low level of protein could be detected in cultured fibroblasts by Western blot (Figure S2). The data demonstrate that wildtype mammalian FIG4 protein is dependent on interaction with VAC14 for stability.

### Generation of Fig4-I41T transgenic mice

**Figure 3:**
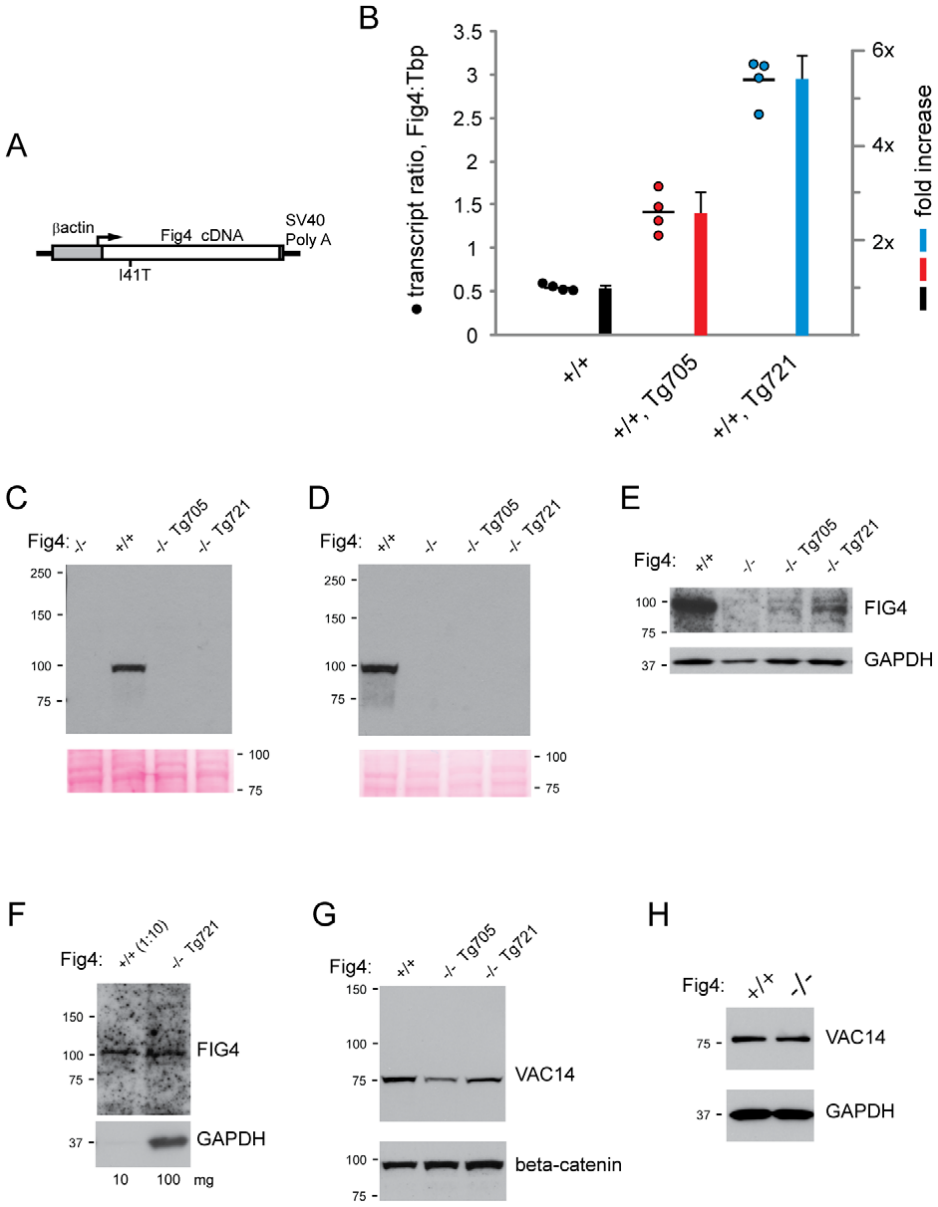
Structure and expression of the *Fig4*-I41T transgene. A) Structure of the *Fig4*-I41T transgene containing the mouse *Fig4* cDNA downstream of the ubiquitously active chicken β-actin promoter. B) Quantitation of transgene mRNA by qRT-PCR using RNA from Fig4^+/+^ wildtype mice with the indicated transgene. RNA was isolated from brain at P28. The ratio of *Fig4* transcript to *Tbp* (Tata binding protein) transcript is calculated as described in Methods. C) Western blots of brain and D) kidney extracts from Tg705 and Tg721 transgenic lines stained with the monoclonal anti-FIG4 antibody and compared with tissues from wildtype and Fig4 null mice. 100 ug of protein, 3 min film exposure. E) FIG4-I41T protein can be detected in 100 ug of brain extract from transgenic lines after film exposure for 30 min. F) FIG4-I41T in 100 ug of brain protein from Tg721 line is comparable to FIG4 in 10 ug of wildtype brain. G, H) VAC14 protein is present in brain from transgenic mice and *Fig4* ^−/−^ null mice.

**Figure 4:**
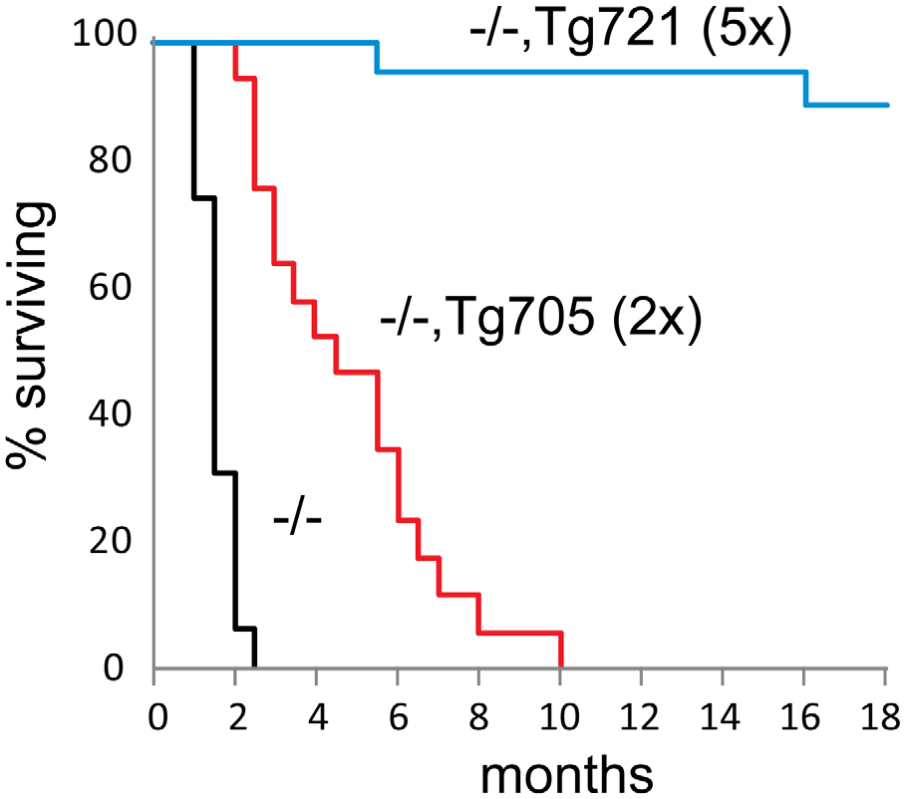
Rescue of juvenile lethality by expression of the Fig4-I41T transgene. Kaplan-Meijer survival curves for *Fig4* null mice carrying Tg705 (n = 17), Tg721 (n = 21) transgenes or lacking the transgene (n = 28). Transgene expression is indicated relative to wildtype expression.

To investigate the *in vivo* function of the mutant protein, we generated a transgene construct containing the mouse *Fig4*-I41T cDNA under the direction of the ubiquitously expressed chicken β-actin promoter (Figure 3A). Expression of *Fig4*-I41T on a wildtype background did not cause any visible abnormality in two independent transgenic lines, which exhibited normal fertility and life span. The transgene copy-number was measured by analysis of genomic DNA by qPCR and demonstrated a transgene copy number of 4 copies in line Tg705 and 2 copies in line Tg721 (Figure S3).

### Quantitation of transgene transcript

Brain RNA was prepared from mice carrying the transgene on a wildtype genetic background. qRT-PCR reactions were carried out with Taqman primers in exon 3 and 4 and the product was detected with a probe spanning the junction between exon 3 and exon 4. Both endogenous transcripts and transgene-derived transcripts are detected by this assay. The abundance of Fig4 transcripts was compared with Tata binding protein (Tbp) transcripts as an internal control (see Materials and Methods). The ratio of *Fig4* transcripts to *Tbp* transcripts in *Fig4* ^+/+^,Tg705 brain was 3× higher than in nontransgenic *Fig4* ^+/+^ mice (Figure 3B). Subtracting the 1× contribution from the endogenous *Fig4* alleles, the contribution of the 705 transgene is 2× the level of endogenous expression. In the brain of the higher expressing line Tg721, *Fig4* transcript was 6× higher than in *Fig4^+/+^* mice, indicating that the transgene transcript is expressed at 5× endogenous expression (Figure 3B).

### Low level of FIG4-I41T protein in transgenic lines on the *Fig4* null background

In order to generate a model of CMT4J expressing I41T in the absence of wildtype *Fig4* protein, the FIG4-I41T transgenic mice were crossed with heterozygous mice carrying the *Fig4* null (plt) allele. *Fig4*-null mice carrying the I41T transgene were generated in the expected Mendelian proportions from standard two-generation crosses with both transgenic lines. When extracts from brain and kidney of *Fig4^−/−^*,Tg705 and *Fig4^−/−^*,Tg721 mice were examined by Western blotting, the abundance of FIG4-I41T protein was substantially lower than in wildtype tissues (Figure 3C, 3D). With longer exposure, a low level of FIG4-I41T protein could be detected in the Tg705 line, and a higher level in the Tg721 line, consistent with their relative transcript levels (Figure 3E). Comparison with a 1∶10 dilution of wildtype brain indicated that the level of FIG4-I41T protein in line Tg721 is approximately 10% of wildtype (Figure 3F). The low abundance of I41T protein was confirmed with a second antibody, a rabbit polyclonal antibody generated to the same C-terminal antigen (Figure S4).

The abundance of VAC14 protein is normal in transgenic and *Fig4* null mice (Figure 3G, 3H). Thus low VAC14 protein is not responsible for the low level of FIG4-I41T protein in the transgenic lines. The data are consistent with the evidence above from VAC14 null mice, and indicate that the FIG4-I41T protein is destabilized *in vivo* by its reduced affinity for VAC14.

### Dose-dependent survival in the I41T transgenic lines

Inheritance of the low expressing transgene Tg705 increased survival of *Fig4* null mice from 1–2 months to 3–6 months (Figure 4). These mice provide a model of human CMT4J, as described below. In the high-expressing Tg721 line, lethality was completely corrected (Figure 4). The oldest cohort of *Fig4^−/−^*,Tg721 mice have survived more than 28 months with no visible abnormalities. The data demonstrate that a relatively low level of Fig4-I41T protein can rescue the lethality of *Fig4* null mice.

### Rescue of neurodegeneration by the *Fig4-I41T* transgene

**Figure 5:**
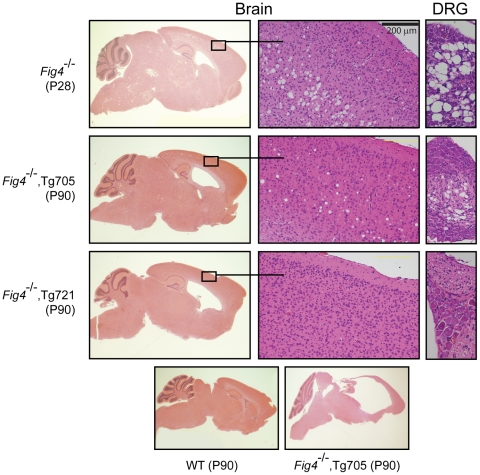
Spongiform degeneration of brain and DRG is rescued by the Fig4-I41T transgene. Top: Sagittal brain section, cortex, and DRG from *Fig4* null mice demonstrating enlarged ventricle and spongiform degeneration (scale bar = 200 µm). Middle panels: Partial rescue of Fig4 null mice carrying Tg705 (2× expression) and Tg721 (5× expression). Bottom panel: Sagittal sections of brain from wildtype mice (left) and transgenic mice in the final stage of disease progression. Both transgenes provide protection from neurodegeneration, with greater protection in the higher expressing line.

*Fig4* null mice exhibit a reproducible pattern of spongiform degeneration in the brain and extensive loss of neurons from peripheral ganglia (Figure 5, top panel). Neurons in layers 4 and 5 of the cortex, the deep cerebellar nuclei, and the dorsal root ganglia (DRG), are severely affected, with accumulation of vacuoles that fill the cytoplasm [1]. In null mice carrying the Tg721 transgene, these abnormalities are almost completely eliminated. Spongiform degeneration of the brain is minimal and DRG neurons are intact at P90 (Figure 5). In line Tg705, an intermediate level of degeneration is visible at P90. In both transgenic lines degeneration of the cerebellar nuclei is visible, indicating that this region is extremely sensitive to PI(3,5)P_2_ levels (Figure S5). Overall, neurodegeneration is rescued in a dose-dependent manner.

Enlarged lateral ventricles and hydrocephalus are seen in both transgenic lines (Figure 5, left panels). High pressure hydrocephalus is indicated by the compression of the cerebellum and hippocampus observed in all of the Fig4^−/−^,Tg705 mice near the end of their lifespan (Figure 5, bottom panel). This is also indicated by the domed appearance of the head and the expulsion of cerebrospinal fluid during dissection. The hydrocephalus in these mice is very similar to that in the L156R (ingls) mutant of *Vac14* [3].

### Rescue of astrocytosis and autophagy in transgenic mice

**Figure 6:**
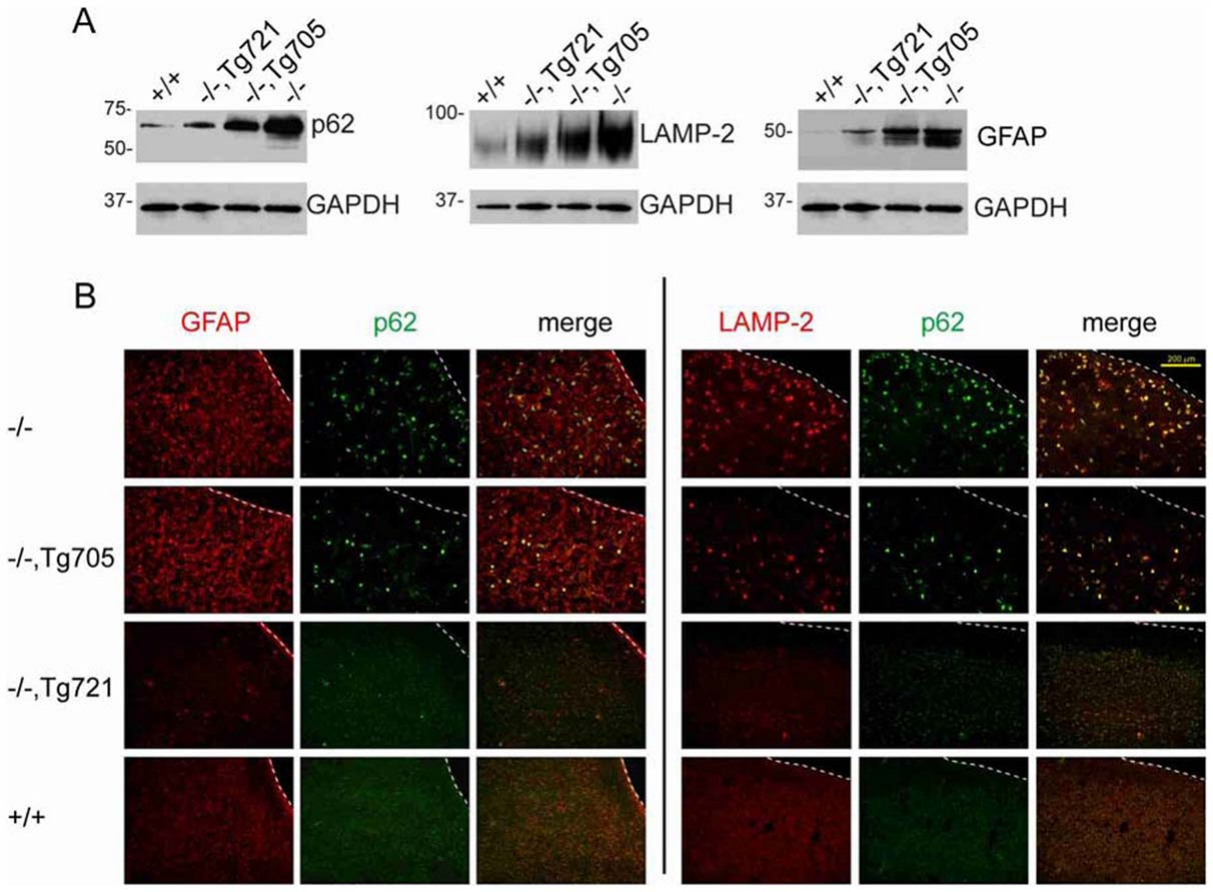
Dose-dependent rescue of the autophagy defect in brain of transgenic mice. A) Western blots of accumulated autophagy markers LAMP2 and p62 and astrocyte marker GFAP in brain homogenates from wildtype, transgenic and *Fig4* null mice at P28; 30 ug protein per lane. B) Accumulation of of LAMP2 and p62 immunofluorescence in GFAP-positive astrocytes in the cortex of transgenic lines at P28. Astrocyte abundance is dramatically increased in *Fig4* null mice (Scale bars = 200 µm).

Spongiform neurodegeneration in *Fig4* null brain is accompanied by accumulation of p62 and other autophagy intermediates, predominantly in activated astrocytes [11]. In brain from transgenic mice, there is an intermediate level of accumulation of the autophagy markers p62 and LAMP-2 (Figure 6A). Accumulation of the astrocyte protein GFAP is also corrected in a dose-dependent manner (Figure 6A). Reduction of astrocytosis in Tg721 mice is indicated by the decreased number of GFAP positive cells (Figure 6B). Accumulated p62 and lysosomal membrane protein LAMP-2 are localized in astrocytes of the trangenic mice (Figure 6B), as previously shown in null mice [1]. In the high expressing Tg721 line, astrocytosis and GFAP accumulation are almost completely corrected (Figure 6B).

### Sciatic nerve myelination is rescued in transgenic mice

**Figure 7:**
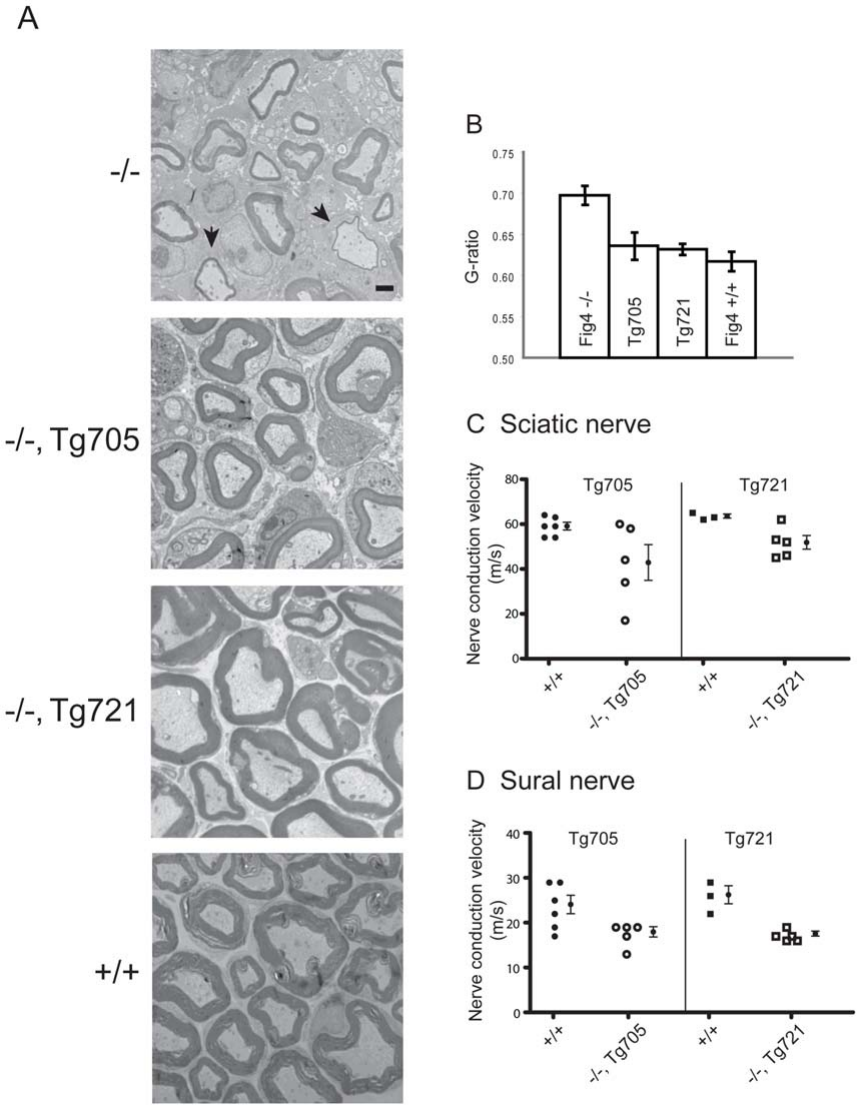
Rescue of peripheral nerve myelination and nerve conduction velocity in transgenic Tg705 and Tg721 lines. A) Transmission electron microscopy of cross-sections of sciatic nerve from *Fig4* mutant and wildtype mice at P21. Arrows in the top panel indicate thinly myelinated axons in *Fig4^−/−^* mice. B) Quantitation of g-ratio in sciatic nerve; higher g-ratio indicates a thinner myelin sheath. *Fig4* ^−/−^ (n = 89 axons); *Fig4* ^−/−^, Tg705 (n = 181 axons); *Fig4* ^−/−^, Tg721 (n = 152 axons); *Fig4* ^+/+^ (n = 109 axons). Scale bar: 5 µm. Error bars, SEM. P<0.05 for Fig4^−/−^ versus WT, Fig4^−/−^ versus Tg705, and Fig4^−/−^ versus Tg712 (Student's t-test). C, D) Nerve conduction velocity was measured in sciatic nerve and sural nerve from 4 month old unaffected Fig4^−/−^,Tg705 mice and 14 month old unaffected Fig4^−/−^,Tg721 mice (mean +/− SEM).

The defective myelination of peripheral nerves characteristic of Charcot-Marie-Tooth disease is also seen in *Fig4* null mice ([1] and Figure 7A, top panel, arrows). In the two transgenic lines, sciatic nerve myelination was comparable to wildtype (Figure 7A). To quantitate axonal myelination we calculated the g-ratio, the inner axon diameter divided by the diameter of the nerve fiber (Figure 7B). At postnatal day 21, the g-ratio of 0.5 for wildtype sciatic nerve is increased to 0.7 for *Fig4* null sciatic nerve by the thinning of the myelin sheath. In the transgenic mice the g-values were restored to wildtype (Figure 7B).

Slowed nerve conduction secondary to defective myelination is another characteristic of Charcot-Marie-Tooth disease that is reproduced in *Fig4* null mice [1]. In sciatic nerve of null mice at 1 month of age, the conduction velocity was 50% of the wildtype value [1]. In contrast, conduction velocities in sciatic and sural nerves from the two transgenic lines, measured at 4 months and 14 months of age, varied between 65% and 80% of wildtype (Figure 7C, 7D), demonstrating partial rescue of the defect. The amplitude of the compound action potential in sciatic nerve was was also restored in the transgenic mice (data not shown).

### Rescue of coat color in transgenic mice

*Fig4*-null mice have diluted pigmentation due to reduced numbers of melanosomes in the mature hair follicle and clumping of melanosomes within the hair shaft [1]. Pigmentation is partially rescued in the higher expressing line Tg721, but not in line Tg705 (Figure S6). In view of the role of *Fig4* in autophagy, it is interesting that autophagy components may play a role in melanosome biogenesis [16].

### Expression of FIG4-I41T in patient fibroblasts

**Figure 8:**
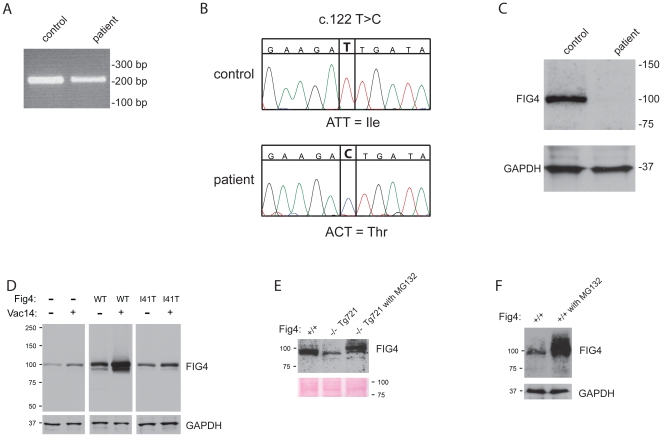
*FIG4* transcript and protein in CMT4J patient fibroblasts. A) RT-PCR products containing exon 2 were amplified from total fibroblast RNA with a forward primer in exon 1 and reverse primer in exon 3. B) Sequence of the RT-PCR product demonstrating nucleotide c.122T (isoleucine) in the control RNA and nucleotide c.122C (threonine) in the patient RNA. C) Western blot of 60 ug of protein from patient and control fibroblasts. D) HEK293T cells transfected with wildtype mouse FIG4 cDNA transgene (Figure 3) or the corresponding I41T construct. Co-transfection of VAC14 stabilizes endogenous and transfected FIG4 proteins. E) The low level of FIG4 in primary fibroblasts from Tg721 transgenic mice is increased by culture for 8 hours in the presence of 10 uM concentration of the proteasome inhibitor MG-132. F) Incubation of wildtype fibroblasts with MG-132 increases the level of FIG4 protein.

In order to determine whether the low abundance of the FIG4-I41T protein in transgenic mice was representative of patient tissues, we examined fibroblasts from a CMT4J patient who is a compound heterozygote for I41T in exon 2 and the null allele R183X in exon 6 [2]. Exon 2 was amplified from patient and control fibroblast RNA (Figure 8A). The sequence of the control RNA contains a T nucleotide at position 122, encoding the wildtype isoleucine allele. The patient RNA contains a C nucleotide encoding threonine (Figure 8B). The absence of the wildtype nucleotide in patient RNA indicates that the R183X transcript is not stably expressed, probably due to degradation by nonsense-mediated decay [17], consistent with its location at a distance from the C-terminal exon 23.

Western blot analysis of fibroblast extracts demonstrated that FIG4-I41T protein in patient fibroblasts is dramatically reduced in comparison with wildtype (Figure 8C). The very low abundance of FIG4-I41T protein, well below the expected 50% of normal, indicates that the mutant protein is unstable in patient tissues, as it is in the transgenic mice.

### Stabilization of FIG4 by co-transfection of VAC14 in HEK293 cells

The level of endogenous FIG4 in HEK293 cells was increased by transfection of VAC14 (Figure 8D, lane 1 and 2). The level of tranfected wildtype mouse FIG4 in HEK cells was also increased by co-transfection of VAC14 (Figure 8D, lane 3 and 4). The abundance of FIG4-I41T after transfection was much lower than for the wildtype protein, and the effect of co-transfected VAC14 was much smaller (Figure 8D, lane 5 and 6). These experiments demonstrate that wildtype FIG4 can be stabilized by coexpression of VAC14 in cultured cells, and that mutant FIG4-I41T is less effectively stabilized.

### Effect of proteasome inhibition on FIG4-I41T

The level of FIG4 protein in primary fibroblasts from Tg721 transgenic mice is lower than in fibroblasts from wildtype mice (Figure 8D), but is sufficient to prevent vacuolization (data not shown). It was recently reported that the proteasome inhibitor MG-132 increases the level of endogenous FIG4 protein in COS7 cells, indicating that there is turnover of FIG4 in the proteasome [14]. To determine whether the mutant FIG4-I41T protein could be stabilized, we cultured primary fibroblasts for 8 hrs with 10 uM MG-132. The level of FIG4-I41T protein was significantly increased by this treatment in fibroblasts from Tg721 transgenic mice (Figure 8E). In control fibroblasts the level of wildtype FIG4 was also increased by culture with MG-132 (Figure 8F). The MW of protein produced in the presence of MG-132 was higher than in untreated cells (Figure 8E, 8F). Since MG-132 does not prevent ubiquitination of substrates, the higher MW may be a consequence of ubiquitination of the FIG4 protein.

## Discussion

We have demonstrated that the FIG4-I41T protein is unstable *in vivo* in cultured cells and in transgenic mice, and that the amount of protein in cells from CMT4J patients is extremely low. FIG4 protein expression equivalent to 10% of wildtype levels is sufficient to prevent neurodegeneration and completely rescue lethality in transgenic line Tg721. In contrast, transgenic line Tg705, with lower expression of FIG4-I41T, provides an animal model of the human disorder with neurodegeneration. These key observations suggest that increasing the expression of the FIG4-I41T allele in CMT4J patients, or stabilizing the protein, would be therapeutic.

The low level of FIG4 protein in rescued mice and in patient fibroblasts appears to be a consequence of the direct effect of the I41T mutation on interaction with the scaffold protein VAC14. In yeast the co-localization of Fig4p, Fab1p and Vac14p on the vacuolar membrane requires the presence of all three proteins, and loss of one protein prevents localization of the other two [3]–[5]. Similarly, in mammalian cells expressing an shRNA to downregulate *Vac14* expression, a small reduction in endogenous FIG4 protein was reported [14].

We confirmed the importance of the FIG4-VAC14 interaction for stability of wildtype FIG4 with the demonstration that FIG4 protein is drastically reduced in mice homozygous for a null allele of VAC14. This experiment clearly demonstrates the dependence of wildtype FIG4 protein on VAC14 for *in vivo* stability. Because of its reduced affinity for VAC14, FIG4-I41T is a hypomorphic allele encoding an unstable protein, resulting in a very low steady-state level of protein *in vivo*. The VAC14 protein is composed of heat-repeat domains [3] and is thought to function as a scaffold for the PI(3,5)P_2_ biosynthetic complex. In another example of the importance of interactions between proteins in this complex, the missense mutation of *Vac14* in the *ingls* mouse which reduces the affinity of VAC14 for FAB1 and results in a neurodegenerative disease that closely resembles the *Fig4* null mice [3]. In the reciprocal experiment, VAC14 protein was *not* reduced in *Fig4* null mice, demonstrating the greater intrinsic stability of the mammalian scaffold protein, and/or its stabilization by interaction with other components of the complex.

**Figure 9:**
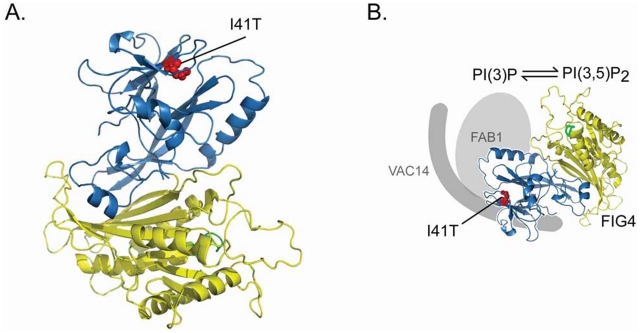
Location of the *FIG4*-I41T mutation and effect on protein interaction. A) Predicted structure for FIG4 phosphatase based on protein coordinates of yeast Sac1 phosphatase(18). The isoleucine residue mutated in CMT4J (red) is located near the surface of the N-terminal domain (blue) that is predicted to function in protein-protein interaction [18]. The catalytic domain (yellow) contains the active site (green). *Image courtesy of Yuxin Mao*. B) Predicted orientation of the FIG4 protein in the PI(3,5)P2 biosynthetic complex [3]. As shown in this paper, the I41T mutation of *FIG4* (red) reduces interaction with both FAB1 and VAC14.

This model of pathogenesis is consistent with the structure of the FIG4 protein, which was predicted by superimposition with the crystal structure of Sac1p, a closely related lipid phosphatase [18]. The I41T mutation is located near the surface of the non-catalytic domain, in a hydrophobic pocket between two β-sheets (Figure 9). The mutation was predicted to affect protein-protein interaction by destabilizing the SacN domain [18], consistent with our observations. The I41T mutation is located at a distance from the catalytic domain, and appears not to affect the enzymatic activity [14].

Based on our observations, increased expression or stabilization of the FIG4-I41T protein in patients with CMT4J should be therapeutic and could achieve complete rescue of this progressive neurodegenerative disorder. Inhibition of proteasome degradation with MG132 resulted in increased levels of FIG4-I41T protein in cultured fibroblasts. This non-specific agent increased the abundance of approximately 200 proteins in cultured fibroblasts [19]. The proteasome inhibitor Velcade (bortezemib) has been approved for treatment of multiple myeloma, and the widely used drug disulfiram (Antabuse) was recently shown to have activity as a proteasome inhibitor [20]. Pharmacological interventions like these might increase FIG4 concentration to a level sufficient to protect against loss of motor function in CMT4J patients with genotype *FIG4^I41T/−^*. Histone deacetylase inhibitors such as sodium butyrate and tricostatin A that increase the expression of other neurological disease genes such as *SMN* should also be evaluated in CMT4J fibroblasts.

The correlated stepwise rescue of autophagy, gliosis, neurodegeneration and lethality in the two transgenic lines supports our proposed model of pathogenesis in which accumulation of autophagy intermediates leads to neuronal damage and then to gliosis, neural cell death and lethality [11], [12]. PI(3,5)P_2_ appears to be required at a step subsequent to the formation of the autolysosome [11], and may be involved in the regeneration of lysosomes from autolysosomes [21]. A newly recognized function of PI(3,5)P_2_ is activation of the lysosomal calcium channel TRPML1, which is mutated in the neurodegenerative disorder mucolipidosis type IV [22]. Transfection of TRPML1 into *Vac14* null fibroblasts rescued the vacuolization caused by deficiency of PI(3,5)P_2_ [22]. Increased lysosomal ion concentrations resulting from PI(3,5)P_2_ deficiency in *FIG4* mutant cells could contribute to vacuolization via osmotic retention of water. Low molecular weight activators of TRPML1 are under development and might provide another route to treatment of PI(3,5)P_2_ deficiency.

The response of various tissues to rescue of FIG4 deficiency in transgenic mice is summarized in Table 1. The dramatically improved survival of cortical and dorsal root neurons, and the complete rescue of lethality, indicate that a small amount of I41T protein is sufficient for normal function in most affected cells.

**Table 1:** Rescue of various phenotypes of the *Fig4* null mice by the Tg705 and Tg721 I41T transgenes.

*Fig4* genotype	−/−	−/−, Tg705	−/−, Tg721	+/+
brain transcript	0	2×	5×	1×
FIG4 protein	none	I41T	I41T	wildtype
survival	1–2 months	3–6 months	>2 years	>2 years
degeneration of cerebral cortex	extensive	intermediate	none	none
degeneration of DRG	extensive	intermediate	none	none
degeneration of deep cerebellar nuclei	extensive	intermediate	intermediate	none
autophagic inclusion bodies	extensive	intermediate	few	none
hydrocephalus	present	present	present	rare
coat color	pale	pale	intermediate	wildtype
nerve conduction velocity	reduced	intermediate	intermediate	normal.
sciatic nerve myelination	defective	normal	normal	normal

Fifteen CMT4J pedigrees segregating the I41T allele have been identified to date ([1], [2] Nicholson et al, unpublished data). In all of the unrelated families the I41T allele is inherited on the same chromosome haplotype, indicating inheritance of a shared founder mutation. In spite of their shared *FIG4^I41T/−^* genotype, the clinical course in CMT4J patients is highly variable. Age of onset ranges from an early childhood form with developmental delays that resembles Dejerine-Sotas syndrome to an adult onset form with rapid progression that may be triggered by trauma [1], [2]. Background genetic variation affecting the level of expression of VAC14 or other proteins in the PI(3,5)P_2_ biosynthetic complex could contribute to the clinical differences in patients with identical *FIG4* genotype. The *Tg705* model of CMT4J, which survives for 3 to 6 months and then develops severe disease, will be useful for testing therapies designed to increase the *in vivo* level of FIG4-I41T protein.

## Materials and Methods

### Ethics statement

This study was carried out in strict accordance with the recommendations in the Guide for the Care and Use of Laboratory Animals of the National Institutes of Health. The protocol was approved by the University Committee on the Use and Care of Animals (UCUCA) of the University of Michigan (Protocol No. 08629).

### *Fig4* null mice

The *Fig4* null mutant “pale tremor” (plt) arose spontaneously in a mixed strain background that included C57BL/6J, C3H, 129 and SJL. This stock was crossed for one generation to strain C57BL/6J. Heterozygous plt/+ offspring were crossed to strain CAST/Ei to produce an F2 generation for genetic mapping [1]. plt/+ F2 mice were intercrossed to initiate a recombinant inbred line that is maintained by brother×sister breeding and is now at generation F12. The genetic background of this line, designated CB.plt, is approximately 50% CAST/Ei and 25% C57BL/6J, with smaller contributions from strains C3H, 129 and SJL.

### Transgenic mice

The mouse *Fig4* transcript was amplified from brain RNA isolated from strain C57BL/6J using two primers containing *SphI* sites: F, ACG CAT GCT ATG CTA TGT GTC TGG TGT GCT GGA GGT CTG and R, TCG CAT GCA GTC CTT TAC CCA TGA GCT GCA TC. The product was digested with *SphI* and subcloned into the corresponding site of the vector pCAG3z [23], [24]. The I41T mutation was incorporated into the Fig4 cDNA clone by site-directed mutagenesis and the construct was completely sequenced. Plasmid DNA was isolated with the Qiagen MaxiPrep Kit and digested to generate a linear fragment containing the cDNA sequence with the promoter and polyadenylation site. Transgenic mice carrying the Fig4-I41T cDNA construct were generated by microinjection of (C57BL/6J X SJL)F2 mouse oocytes at the Transgenic Animal Model Core at the University of Michigan (www.med.umich.edu/tamc). Transgenes were maintained in heterozygous state during breeding to prevent unequal recombination between multi-copy inserts.

### Yeast functional tests

The yeast two-hybrid test and immunoprecipitation analysis were carried out as previously described [3]. Human and yeast *VAC14* were subcloned into the *Cla*I–*Bgl*II and *Xma*I–*Sal*I sites of pGAD, respectively. Human and yeast wild type and mutant *FIG4* were subcloned into the *Xma*I–*Sal*I and *Bam*HI–*Pst*I sites of pGBD, respectively. pGAD and pGBD plasmids were cotransformed into the yeast strain PJ69-4A. Transformants were initially plated onto SC-LEU-TRP media for plasmid selection and replica-plated onto selective plates with SC-LEU-TRP-ADE-HIS+3AT media or SC-LEU-TRP-ADE-HIS media and grown at 24°C for 4 to 14 days for colony formation. The yeast strain PJ69-4A and the pGAD and pGBD vectors have been described [25]. The Venus-derivative of GFP was previously described [15]. Selective media contained 3-amino-1,2,4-triazole (3AT), an inhibitor of His3p.

### Immunofluorescence and Western blots

Immunocytochemistry of p62, LAMP2 and GFAP on fresh frozen cryosections of mouse brain and Western blotting of brain extracts were carried out as previously described [11]. For immunoblotting of FIG4, tissues were homogenized in 0.25 M sucrose, 0.05 M Tris, pH 7.5 and the 21,000× g soluble fraction was analyzed. Immunofluorescence images were captured on an Olympus BX51 microscope equipped with epifluorescence and processed and merged with Adobe Photoshop software.

### FIG4 antibodies

A rabbit polyclonal antiserum and a mouse monoclonal antibody were generated to a C-terminal fragment of FIG4 containing the final 220 amino acids, residues 688 to 907, which is encoded by exons 18 to 23. A 660 bp cDNA fragment was amplified from a full length *Fig4* cDNA derived from strain C57BL/6J and cloned into the XhoI and EcoRI sites of the expression vector pRSETA (Invitrogen Corporation, Calrsbad, CA, USA), adding a polyhistidine tag (6× His) to the N-terminus. Recombinant protein was expressed in *E. coli* and purified from inclusion bodies using Novagen BugBuster Protein Extraction Reagent (EMD Chemicals, Gibbstown, NJ, USA). Rabbit polyclonal antiserum to the purified protein was generated and affinity-purified by Pocono Rabbit Farm and Laboratory, Inc. (Canadensis, PA). The purified polyclonal antiserum was used at 1∶50 dilution for western blots. The monoclonal antibody was generated using the same protein fragment at the UC Davis/NIH NeuroMab Facility (Clone N202/7). The monoclonal was purified from cell culture and used at 1∶200 for western blots. A broad crossreacting band of 50 to 55 kDa is ocassionally observed on Western blots of control and mutant tissues but there is no consistent association with genotype.

### Histology

Brain and spinal cord were fixed for 24 hours at 4°C in phosphate buffered 10% formalin and then in 70% ethanol for an additional 24 hours at 4°C. Paraffin embedding, decalcification of spine, and H&E staining were carried out at Histoserv Inc (Maryland). Images were obtained with an Olympus BX51 microscope and DP50 camera. Image capture settings were identical for sections from −/− and −/−,Tg705 mice that exhibited immunostaining for autophagy markers. To detect the outlines of brain sections from −/−,Tg721 and +/+ mice that lacked immunostaining, a longer image capture time was used.

### qRT-PCR of *Fig4* transcripts

Five ug aliquots of total RNA from whole brain was treated with DNAse I (Invtrogen) and cDNA was prepared using the SuperScript First Strand Synthesis System for RT-PCR (Invitrogen). The Fig4 transcript was quantitated using TaqMan gene expression probe Mm00506074_m1 (ABI) which spans the junction between exon 3 and exon 4. As an internal control, the TATA binding protein (Tbp) transcript was quantitated using TaqMan probe Mm00446971_m1. Fluorescence was measured on a Step One Real Time PCR System (ABI) at the Microarray Core at the University of Michigan. A linear relationship between copy number and C_T_, the threshold cycle for detection of fluorescence, was observed (Figure S3). The mean C_T_ value was determined from quadruplicate assays of each sample. The value of ΔC_T_ was calculated by subtracting the C_T_ for *Tbp* from the C_T_ for *Fig4*. The ratio of *Fig4* transcripts to *Tbp* transcripts was calculated as 2^−ΔCT^.

### Transgene copy number determination by qPCR

Transgene copy number was determined using the TaqMan probe described above. A standard curve was generated using pCAG3Z-Fig4 diluted into wildtype mouse DNA for copy-number standards of 0, 0.5, 1, 2, 4, 8, and 16 copies per haploid genome using 2.83 pg/ug for one haploid copy equivalent for this 3 kb transgene. Genomic DNA (50 ng) from Tg705/+ mice, Tg721/+ mice, and standards were assayed in quadruplicate. The Taqman probe includes the junction between exon 3 and exon 4, which are separated by 4 kb in genomic DNA. No fluorescent signal was obtained from 8 replicates of wildtype genomic DNA. R^2^ for the standard curve was 0.999. Observed copy numbers for both transgenic lines are within the linear range of the assay.

### Primary fibroblasts and HEK293 cells

HEK293 cells (passage 15) were maintained in DMEM/F-12 supplemented with 10% fetal bovine serum, penicillin, streptomycin and amphotericin. Subconfluent cells in 10 cm dishes were transfected with 6 ug plasmid DNA and 18 uL FuGene (Roche) for 24 hours in complete culture media. Cells were lysed in RIPA buffer and lysates were spun at 15,000× g for 10 minutes. Supernatant protein was quantitated using the BCA kit (Pierce). Mouse fibroblasts were isolated from P0 mouse tail biopsy by digestion with collagenase type 2 (Worthington labs) and cultured in RPMI 1640 supplemented with 15% fetal bovine serum and containing penicillin, streptomycin and amphotericin. Experiments were carried out at passage 3 to 5. Treatment with 10 uM MG-132 (Sigma) was carried out in complete media for 8 hours. Primary cultured fibroblasts from CMT4J patient 2 were previously described [8]; passage 4 cells were kindly provided by Dr. Jun Li, Department of Neurology, Vanderbilt University.

### Nerve conduction velocity

Nerve conduction velocity (NCV) was measured as previously described [26]. Mice were anesthetized and maintained at a 34°C core temperature with a heating lamp. Sural sensory NCV was determined by recording at the dorsum of the foot and antidromically stimulating with supramaximal stimulation at the ankle. NCV was calculated by dividing the distance by the take-off latency of the sensory nerve action potential. Sciatic-tibial motor NCV was determined by recording at the dorsum of the foot and orthodromically stimulating with supramaximal stimulation first at the ankle, then at the sciatic notch. Latencies were measured in each case from the initial onset of the compound muscle action potential. The sciatic-tibial motor NCV was calculated by subtracting the measured ankle distance from the measured notch distance. The resultant distance was then divided by the difference in the ankle and notch latencies for a final nerve conduction velocity.

### Electron microscopy of sciatic nerve axons

Mice were anesthetized with ketamine and xylazine and perfused transcardially with 3% paraformaldehyde (Electron Microscopy Sciences) and 2.5% glutaraldehyde (Ted Pella, Inc.). Sciatic nerves were dissected and post-fixed for several hours in perfusion solution at 4°C. Tissues were incubated in a 1% solution of OsO_4_ and embedded in epoxy resin. Ultrathin (75 nm) sections were cut and visualized with a Philips CM-100 TEM. Images were analyzed at 2600-fold magnification using Image-J software for quantification of g-ratio (defined as the diameter of an axon within its myelin sheath divided by the diameter of the axon outside of its myelin sheath). Two measurements were made per axon in order to account for elongated or irregular shape, and the mean was used. Only myelinated axons were used in for the calculation of g-ratio. Data were compared using Microsoft Excel.

### Accession numbers

Accession numbers for the genes referred to in this paper are: human FIG4 (Entrez Gene ID 9896), mouse Fig4 (Entrez Gene ID 103199), yeast Fig4p (Entrez Gene ID 855392), mouse Vac14 (Entrez Gene ID 234729), yeast Vac14p (Entrez Gene ID 851102), mouse Fab1/PIKfyve (Entrez Gene ID 18711), yeast Fab1p (Entrez Gene ID 850574).

## Supporting Information

Figure S1 Specificity of the monoclonal anti-FIG4 antibody. A)The indicated 220 amino acid C-terminal fragment of FIG4 (immunogen) was isolated after bacterial expression and provided to the UC Davis/NIH NeuroMab Facility for generation of the monoclonal antibody. B) Antibody from NeuroMab (Clone N202/7) was diluted 1∶200 for immunostaining of Western blots containing 100 ug of protein from brain homogenate. Equal protein transfer to the filter for wildtype and *Fig4* null brain is demonstrated by Ponceau Red staining.
(TIF)

Figure S2 Low level of wildtype FIG4 protein in cultured fibroblasts from *Vac14* null mice. The Western blot was probed with the monoclonal anti-FIG4 antibody. FIG4 protein could not be detected in P0 tissue, as shown in the text; the level of FIG4 in cultured fibroblasts is just above the detectable level. Each lane contained 60 ug of protein. Comparable loading of the two lanes is demonstrated by the Ponceau-stained gel below. The low abundance of FIG4 protein in the *Vac14* null mouse demonstrates the importance of the VAC14 scaffold protein for stabilization of wildtype FIG4 *in vivo*.
(TIF)

Figure S3 Transgene copy number in two lines of Fig4-I41T transgenic mice. Copy number was assessed by quantitative PCR of genomic DNA. Standards were prepared by addition of varying amounts of transgene plasmid DNA to wildtype genomic DNA. The linear relationship between copy number and the threshold cycle number CT is demonstrated at the right. The PCR primers are located in exon 3 and exon 4 and do not amplify the endogenous *Fig4* gene because the exons are separated by a 4 kb intron in genomic DNA.
(TIF)

Figure S4 Western blot with polyclonal rabbit anti-FIG4 demonstrates low abundance of FIG4-I41T protein in transgenic lines. The polyclonal antibody was generated against the bacterially-expressed 220 amino acid C-terminal protein fragment used to generate the monoclonal antibody (Figure S1). Each lane contains 100 ug of brain soluble protein.
(TIF)

Figure S5 Incomplete rescue of cerebellar nuclei in Fig4-I41T transgenic mice. (Scale bar = 500 µm).
(TIF)

Figure S6 Partial rescue of the pigmentation defect in transgenic mice. The diluted pigmentation of congenic B6.Tg705 and B6.Tg721 mice compared with wildtype black mice (+/+). The (C57BL/6J X SJL)F2 transgenic founders were backcrossed to strain C57BL/6J to generate the B6.Tg705 and B6.Tg721 lines. Transgenic B6 mice at N6 were crossed with the congenic line B6.plt (N10) to generate the null transgenic mice shown here.
(TIF)
