# Supplementary figures and images for: Pathogenic Mechanism of the FIG4 Mutation Responsible for Charcot-Marie-Tooth Disease CMT4J
Source: PLoS Genet. 2011 Jun 2;7(6):e1002104. doi: 10.1371/journal.pgen.1002104 (PMC3107197; doi:10.1371/journal.pgen.1002104)

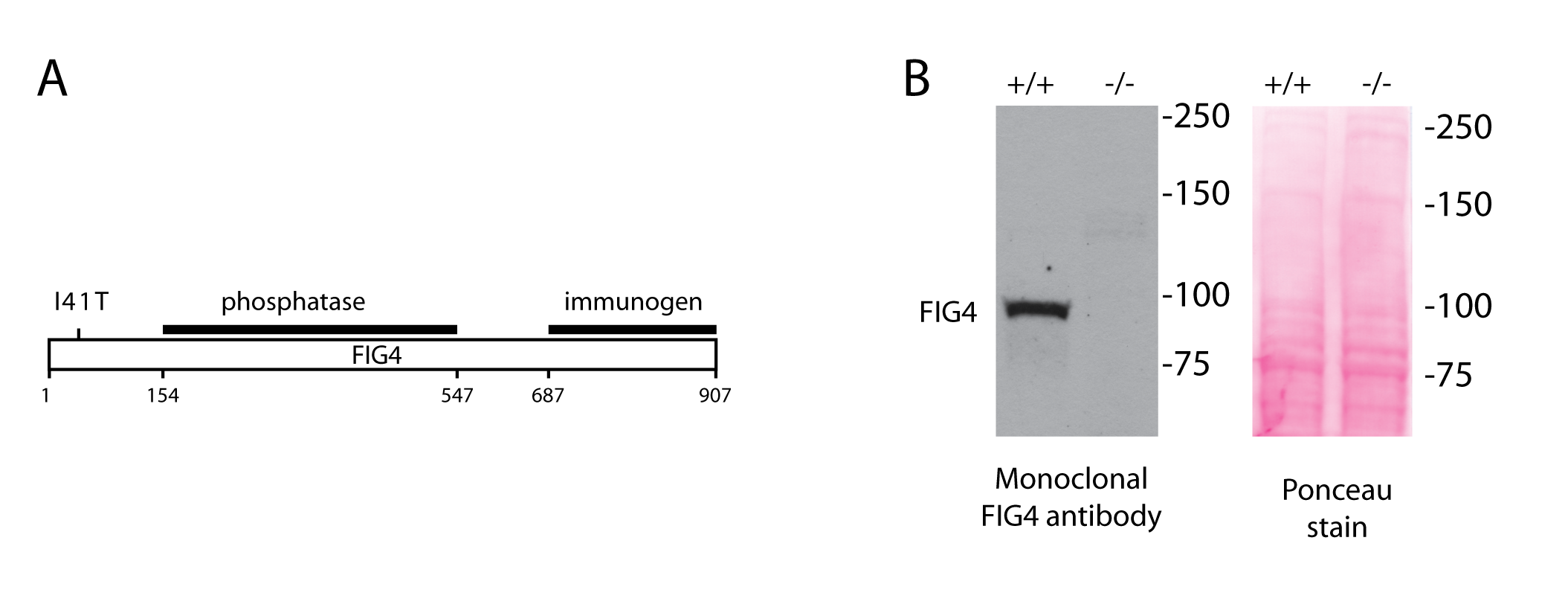

Supplement: Figure S1 — Specificity of the monoclonal anti-FIG4 antibody. A)The indicated 220 amino acid C-terminal fragment of FIG4 (immunogen) was isolated after bacterial expression and provided to the UC Davis/NIH NeuroMab Facility for generation of the monoclonal antibody. B) Antibody from NeuroMab (Clone N202/7) was diluted 1∶200 for immunostaining of Western blots containing 100 ug of protein from brain homogenate. Equal protein transfer to the filter for wildtype and Fig4 null brain is demonstrated by Ponceau Red staining. (TIF) [file pgen.1002104.s001.tif]

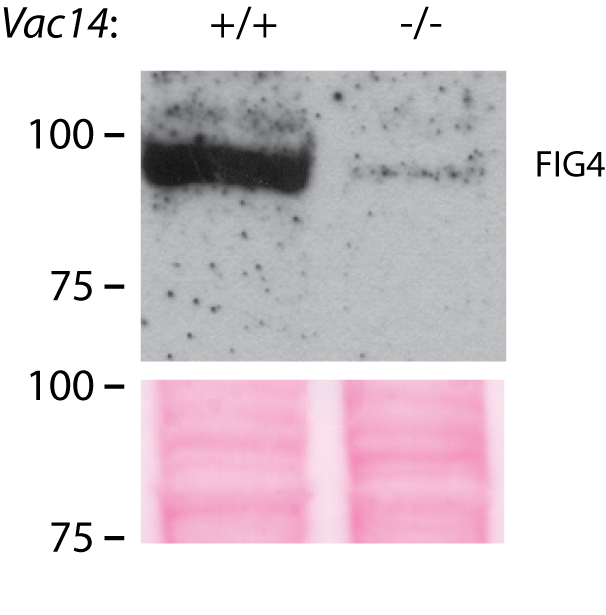

Supplement: Figure S2 — Low level of wildtype FIG4 protein in cultured fibroblasts from Vac14 null mice. The Western blot was probed with the monoclonal anti-FIG4 antibody. FIG4 protein could not be detected in P0 tissue, as shown in the text; the level of FIG4 in cultured fibroblasts is just above the detectable level. Each lane contained 60 ug of protein. Comparable loading of the two lanes is demonstrated by the Ponceau-stained gel below. The low abundance of FIG4 protein in the Vac14 null mouse demonstrates the importance of the VAC14 scaffold protein for stabilization of wildtype FIG4 in vivo. (TIF) [file pgen.1002104.s002.tif]

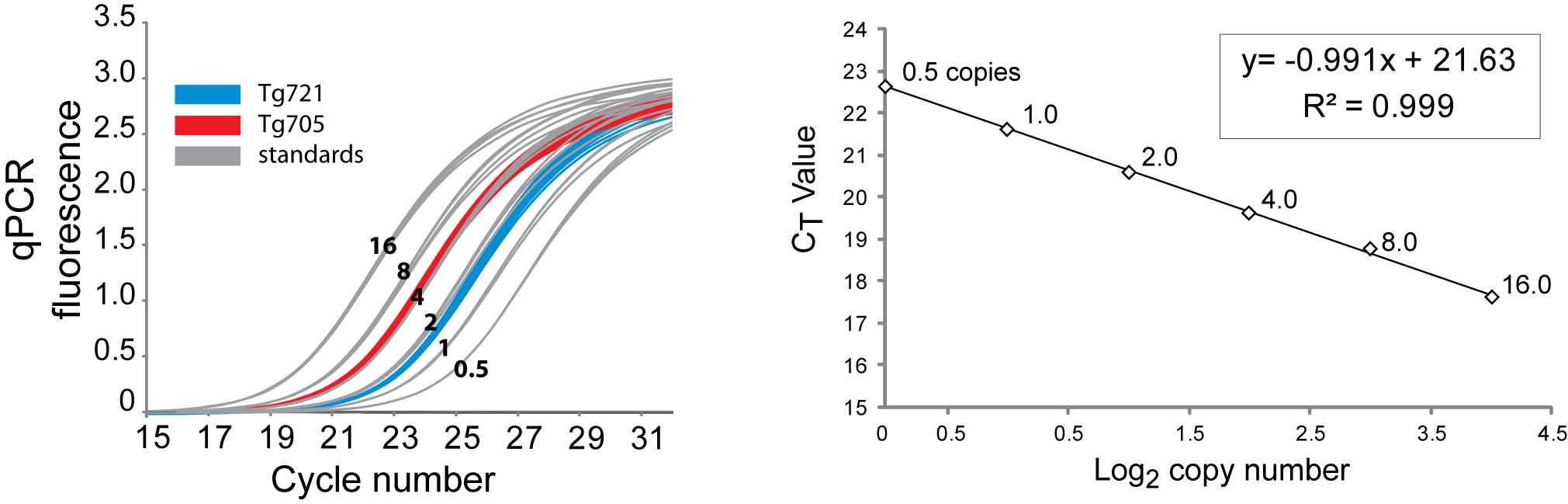

Supplement: Figure S3 — Transgene copy number in two lines of Fig4-I41T transgenic mice. Copy number was assessed by quantitative PCR of genomic DNA. Standards were prepared by addition of varying amounts of transgene plasmid DNA to wildtype genomic DNA. The linear relationship between copy number and the threshold cycle number CT is demonstrated at the right. The PCR primers are located in exon 3 and exon 4 and do not amplify the endogenous Fig4 gene because the exons are separated by a 4 kb intron in genomic DNA. (TIF) [file pgen.1002104.s003.tif]

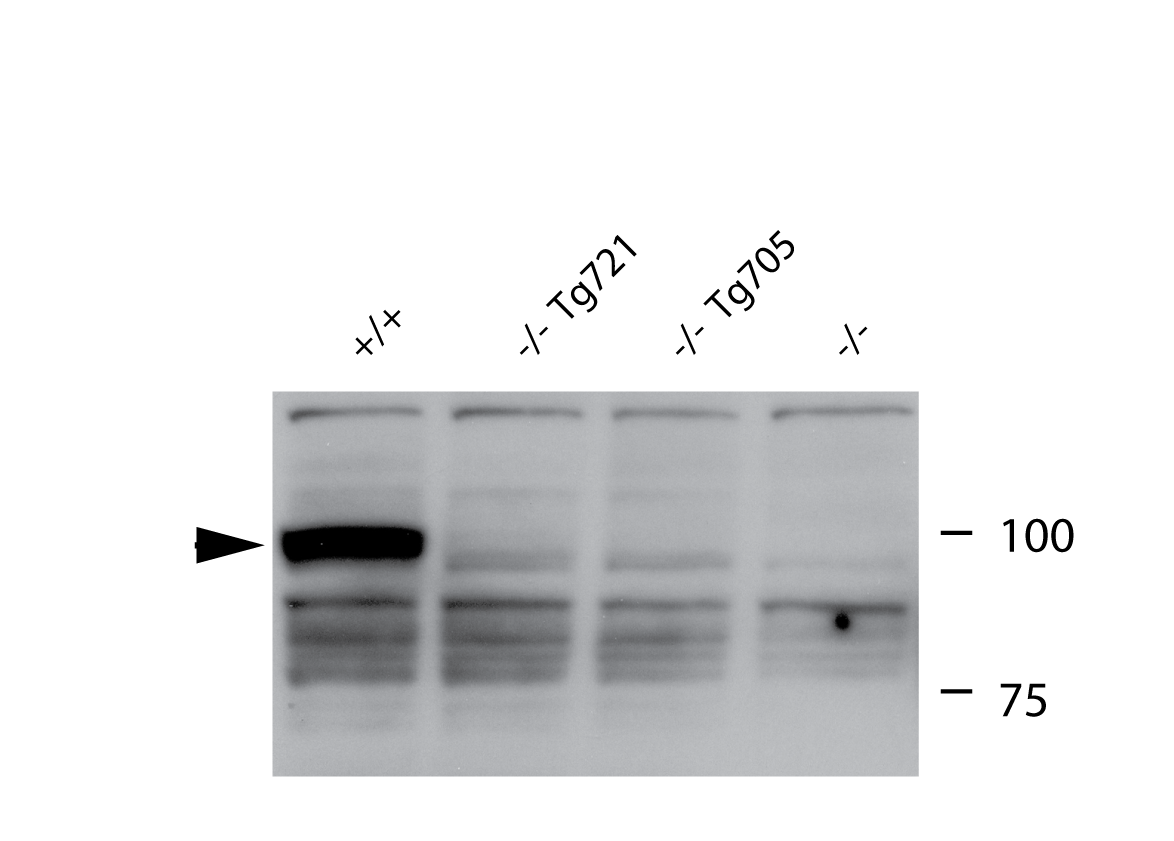

Supplement: Figure S4 — Western blot with polyclonal rabbit anti-FIG4 demonstrates low abundance of FIG4-I41T protein in transgenic lines. The polyclonal antibody was generated against the bacterially-expressed 220 amino acid C-terminal protein fragment used to generate the monoclonal antibody (Figure S1). Each lane contains 100 ug of brain soluble protein. (TIF) [file pgen.1002104.s004.tif]

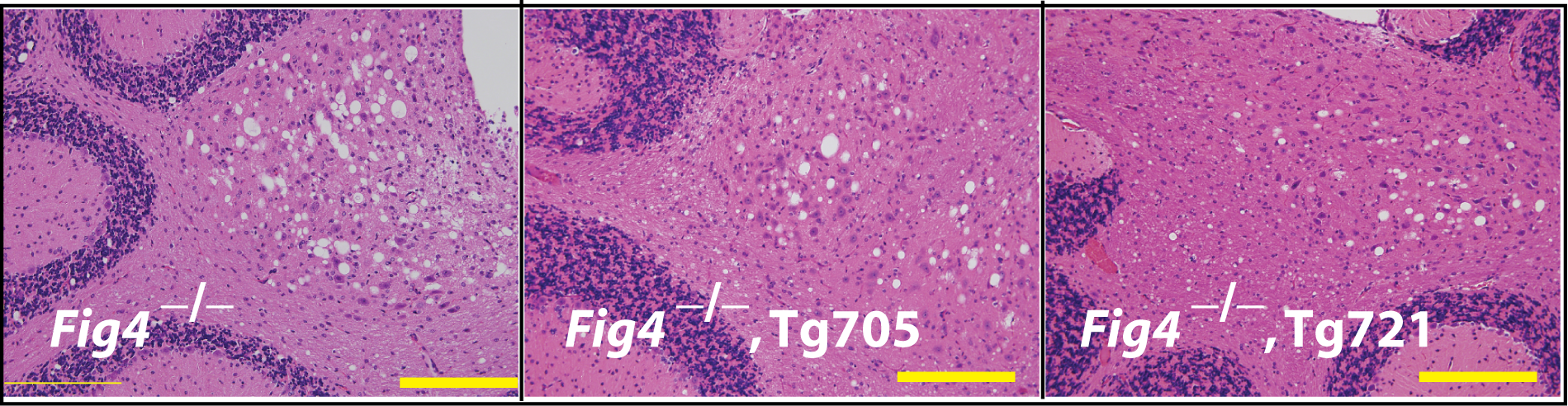

Supplement: Figure S5 — Incomplete rescue of cerebellar nuclei in Fig4-I41T transgenic mice. (Scale bar = 500 µm). (TIF) [file pgen.1002104.s005.tif]

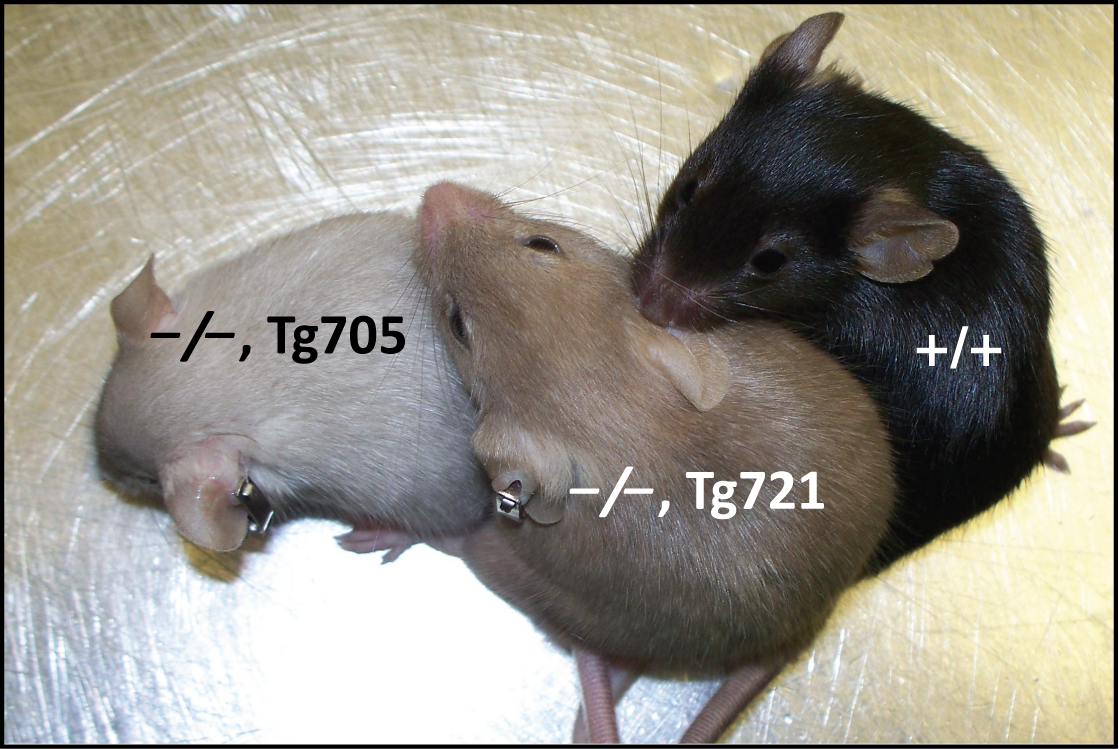

Supplement: Figure S6 — Partial rescue of the pigmentation defect in transgenic mice. The diluted pigmentation of congenic B6.Tg705 and B6.Tg721 mice compared with wildtype black mice (+/+). The (C57BL/6J X SJL)F2 transgenic founders were backcrossed to strain C57BL/6J to generate the B6.Tg705 and B6.Tg721 lines. Transgenic B6 mice at N6 were crossed with the congenic line B6.plt (N10) to generate the null transgenic mice shown here. (TIF) [file pgen.1002104.s006.tif]
